# Comparison of short-term surgical outcomes using da Vinci S, Si and Xi Surgical System for robotic gastric cancer surgery

**DOI:** 10.1038/s41598-021-90741-2

**Published:** 2021-05-26

**Authors:** Toshiyasu Ojima, Masaki Nakamura, Keiji Hayata, Junya Kitadani, Akihiro Takeuchi, Hiroki Yamaue

**Affiliations:** Second Department of Surgery, School of Medicine, Wakayama Medical University, 811-1, Kimiidera, Wakayama 641-8510 Japan

**Keywords:** Gastroenterology, Oncology

## Abstract

When compared with the second/third generation da Vinci S/Si, the fourth generation Xi surgical system may allow for greater efficiency and result in shorter operation times during robotic gastrectomy (RG) for gastric cancer (GC). We directly compare surgical outcomes between the conventional S/Si and the newer Xi robotic platform for the treatment of GC. This is a single-center retrospective study of 148 consecutive patients with GC who underwent RG. Of these patients, 20 patients were treated with the S, 30 patients with the Si, and 98 patients with the Xi. The overall operation time was significantly longer in the S group (386.5 min) than in the other groups (Si group: 292 min; Xi group: 297 min) (S vs. Si: *P* = 0.010; S vs. Xi: *P* = 0.001). We observed no difference, however, between the newer Xi and Si systems in operation time. Intraoperative blood loss was similar across the three groups. The overall postoperative complication rate in the Xi group (8.2%) was lower than that of the S group (10%) and the Si group (13.3%), but the difference was not significant. The newer Xi system did not provide significant intraoperative or early postoperative advantages over the Si system.

## Introduction

Robotic gastrectomy (RG) for gastric cancer (GC) was introduced specifically to overcome the kinematic restrictions of conventional laparoscopic gastrectomy (LG), including the limited range of movement, amplification of operator hand tremors, and inconvenient surgical positioning. We and others have suggested that robotic approach for GC led to a reduction of the incidence of postoperative complications, resulting in improved safety and effective surgical results compared with LG^1–4^. The total operation time, however, has been reported in several studies to be longer by RG than by LG^4–7^. These longer operation times might be attributed to the setting and docking of the robotic arms.

The currently most widely used surgical robotic system is the da Vinci Surgical System (Intuitive, Sunnyvale, CA, USA). The da Vinci Xi is the fourth generation platform, promising easier docking, wider range of motion owing to its thinner arms, improved vision and easier maneuverability^8,9^. We hypothesize that when compared to the second/third generation da Vinci S/Si, use of the Xi can result in greater efficiency and result in shorter operation times. Although there is a study comparing the da Vinci Xi with the da Vinci Si in relation to gastric cancer surgery^10^, data comparing the three robotic systems are still lacking. We therefore directly compare surgical outcomes between the conventional da Vinci S/Si and the newer Xi robotic platform for the treatment of GC.

## Results

### Patient demographics and tumor characteristics

Included in the study were 148 patients who underwent RG. Twenty patients were treated with the da Vinci S Surgical System, 30 patients with the Si system, and 98 patients with the Xi system.

Table 1 shows the patient demographics, including gender, age, body mass index, American Society of Anesthesiologists score, comorbidities, history of abdominal surgery, smoking, and alcohol. No significant difference was observed between the three groups. There were no differences between the three groups in terms of tumor location, histologic type, or distribution of tumor stages (Table 1). Patients treated with the S system had smaller tumors than those treated with the Si or the Xi (*P* = 0.001, Table 1).

**Table 1:** Patient demographics and tumor characteristics.

Variables	da Vinci S (n = 20)	da Vinci Si (n = 30)	da Vinci Xi (n = 98)	*P*
**Patients demographics**				
Gender, Male/Female	14/6	22/8	58/40	0.301
Age, year, median (range)	73 (49–87)	70 (42–876)	72.5 (34–90)	0.440 (S vs. Si)
				0.786 (Si vs. Xi)
				0.517 (Xi vs. S)
BMI, kg/m^2^, median (range)	21.25 (17.4–25.5)	22.1 (15.4–32.1)	21.9 (14.0–30.5)	0.143 (S vs. Si)
				0.687 (Si vs. Xi)
				0.140 (Xi vs. S)
ASA score, 1/2/3	5/14/1	11/18/1	33/61/4	0.937
**Comorbidity**				
Hypertension (%)	11 (55.0)	17 (56.7)	45 (45.9)	0.507
Diabetes (%)	6 (30.0)	6 (20.0)	16 (16.3)	0.358
Pulmonary (%)	0 (0)	2 (6.7%)	10 (10.2)	0.297
Cardiovascular (%)	3 (15.0)	3 (10.0%)	12 (12.2)	0.868
Renal (%)	0 (0)	0 (0)	4 (4.1)	0.350
Hepatic (%)	0 (0)	0 (0)	2 (2.0)	0.596
Cerebrovascular (%)	0 (0)	0 (0)	6 (6.1)	0.203
History of abdominal surgery, (%)	5 (25.0)	5 (16.7)	28 (28.6)	0.425
Open cholecystectomy	0	1	1	
Laparoscopic cholecystectomy	0	1	2	
Appendectomy	2	4	13	
Colorectal surgery	0	0	5	
Gynecological surgery	2	0	9	
Hepatectomy	0	0	1	
Nephrectomy	1	0	0	
Smoking history, Brinkman index, median (range)	25 (0–1500)	329.5 (0–4000)	300 (0–3000)	0.342 (S vs. Si) 0.522 (Si vs. Xi)
				0.550 (Xi vs. S)
Daily drinker (%)	8 (40.0)	16 (53.3)	37 (37.8)	0.314
**Tumor characteristics**				
Location, U (%)/M/L/W	6 (30.0)/5 (25.0)/9 (45.0)/0 (0)	8 (26.7)/5 (16.7)/16 (53.3)/1 (3.3)	27 (27.6)/29 (30.0)/39 (39.8)/3 (3.1)	0.791
Size, mm, median (range)	21 (4–45)	37.5 (20–90)	35 (8–150)	0.001 (S vs. Si)
				0.972 (Si vs. Xi)
				0.001 (Xi vs. S)
Histological type^a^, differentiated (%)/undifferentiated	15 (75.0)/5 (25.0)	18 (60.0)/12 (40.0)	52 (53.1)/46 (46.9)	0.185
pStage^b^, I (%)/II/III/IV	18 (90.0)/2 (10.0)/0 (0)/0 (0)	20 (66.7)/7 (23.3)/3 (10.0)/0 (0)	58 (59.2)/25 (25.5)/11 (11.2)/4 (4.1)	0.207

*BMI* body mass index; *ASA* American Society of Anesthesiologists; *U* upper third of the stomach; *M* middle third of the stomach; *L* lower third of the stomach; *W* whole stomach.

^a^Japanese Classification of Gastric Carcinoma.

^b^UICC 8th edition.

### Converted cases

None of the patients treated with the S system had a converted approach. Two of 30 patients (6.7%) treated with the Si system converted from robotic surgery to laparoscopic surgery, and one of 98 patients (1.0%) converted from robotic surgery to open surgery. Conversion rate was not significantly different between the three groups (*P* = 0.125, Table 2). Detailed characteristics of the three patients are listed in Table 2. Conversion to laparoscopic surgery in the Si group was due to machine trouble in two cases. Conversion to open surgery in the Xi group was due to a technical issue in one case.

**Table 2:** Converted cases.

Group^a^	Gender	Age	Converted approach	Reason for conversion	Procedure	Operation time (min)	Bleeding (mL)	Postoperative complication
da Vinci Si (6.7%)	Male	68	Lap	Machine trouble	TG	412	30	None
da Vinci Si	Male	66	Lap	Machine trouble	DG	259	85	None
da Vinci Xi (1.0%)	Male	78	Open	Portal vein injury	DG + SP	598	2540	None

*Lap* laparoscopic surgery; *Open* open surgery; *TG* total gastrectomy; *DG* distal gastrectomy; *DG* + *SP* distal gastrectomy with en-mass removal of the spleen and body and tail of the pancreas.

^a^*P* = 0.125.

### Surgical results

**Figure 1:**
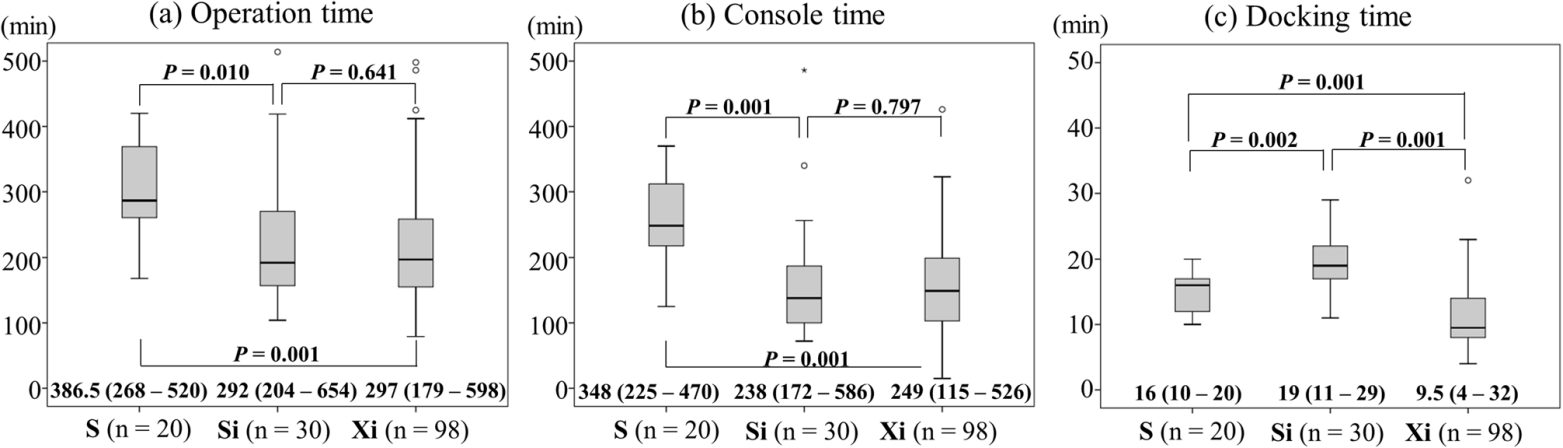
(**a**) Operation time, (**b**) console time and (**c**) docking time. Quantitative results are expressed as medians and ranges. The bars in the figure are expressed as means ± SD. *P* values were calculated with Mann–Whitney U test. S, da Vinci S; Si, da Vinci Si; Xi, da Vinci Xi.

**Figure 2:**
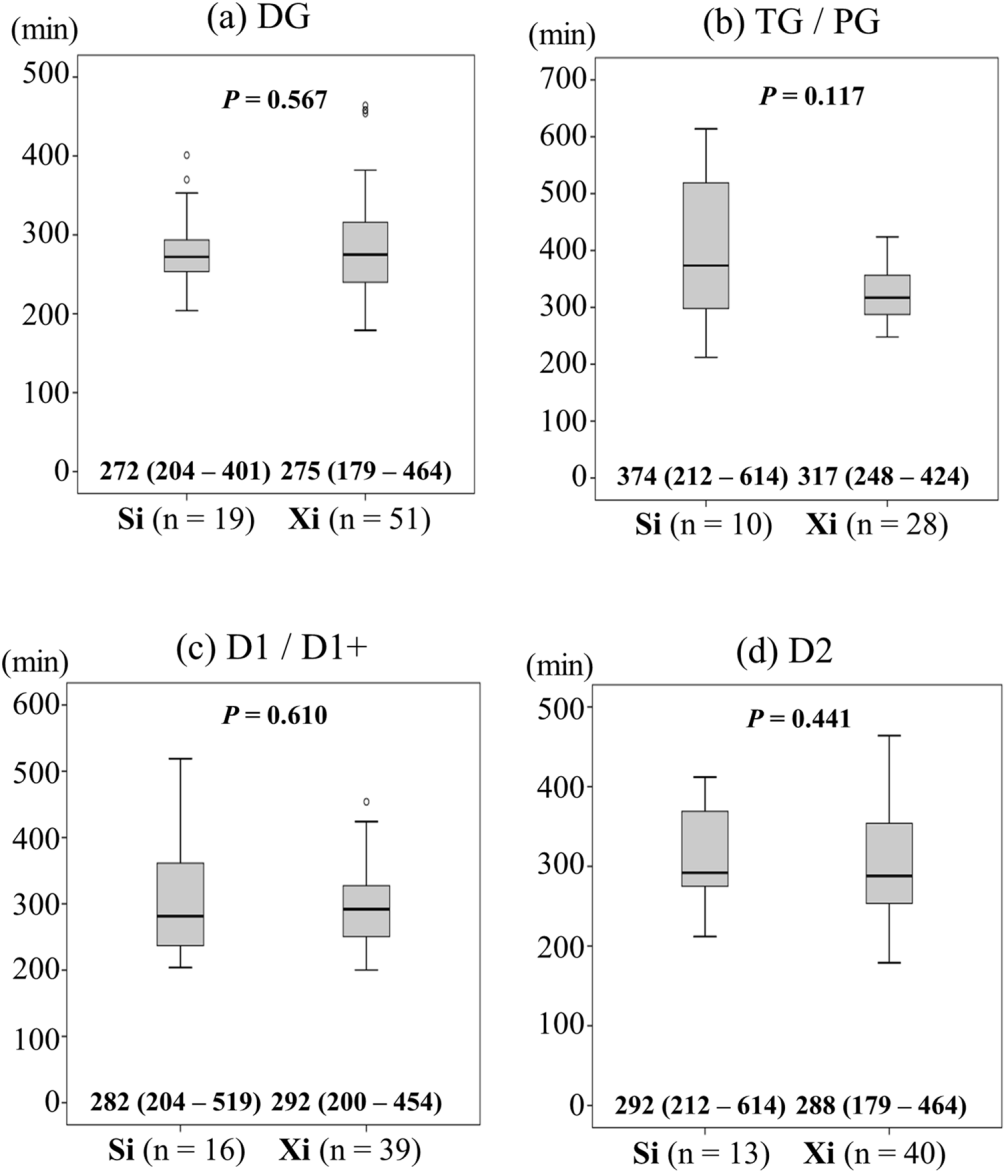
Subgroup analyses of operation time. (**a**) Distal gastrectomy group, (**b**) total and proximal gastrectomy group, (**c**) D1 and D1 + lymph node dissection group and (**d**) D2 lymph node dissection group. Quantitative results are expressed as medians and ranges. The bars in the figure are expressed as means ± SD. *P* values were calculated with Mann–Whitney U test. Si, da Vinci Si; Xi, da Vinci Xi.

Surgical results were also stratified to the three surgical groups (Table 3). There were no significant differences between the three groups in terms of distal gastrectomy to total or proximal gastrectomy ratio, or the range of lymphadenectomy. There was no bias between the two groups regarding the reconstruction procedure. The rates of simultaneous combined resection were significantly higher in the Xi group (19.4%) than in the other groups (S group: 0%; Si group: 3.3%) (*P* = 0.013). Operation time, console time and docking time are shown in Fig. 1. The operation time was significantly longer in the S group (386.5 min) than in the other groups (Si group: 292 min; Xi group: 297 min) (S vs. Si: *P* = 0.010; S vs. Xi: *P* = 0.001). The console time was significantly longer in the S group (348 min) than in the other groups (Si group: 238 min; Xi group: 249 min) (S vs. Si: *P* = 0.001; S vs. Xi: *P* = 0.001). The docking time was significantly shorter in the Xi group (9.5 min) than in the other groups (S group: 16 min; Si group: 19 min) (Xi vs. S: *P* = 0.001; Xi vs. Si: *P* = 0.001). In operation time, there was no significant difference between the Si group (292 min) and the Xi group (297 min) (*P* = 0.641, Fig. 1a). Subgroup analyses by operative procedure or lymph node dissection were performed on patients without simultaneous combined organ resection to clearly confirm that there is no statistically significative difference in operation time between the Si (n = 29) and the Xi (n = 79) groups. As shown in Fig. 2, operation time for Si group and Xi group was also similar in all subgroups. The intraoperative blood loss was similar across the three groups (Table 3). There was no significant difference in the number of retrieved lymph nodes between the three groups, and surgical curability was also similar (Table 3).

**Table 3:** Surgical results and postoperative complications.

Variables	da Vinci S (n = 20)	da Vinci Si (n = 30)	da Vinci Xi (n = 98)	*P*
**Surgical results**				
Operative procedure, DG (%)/TG/PG	14 (70.0)/5 (25.0)/1 (5.0)	19 (63.3)/9 (30.0)/2 (6.7)	59 (60.2)/30 (30.6)/9 (9.2)	0.925
Lymph node dissection^a^, D1 (%)/D1 + /D2/D2 + PAND	0 (0)/16 (80.0)/4 (20.0)/0 (0)	0 (0)/16 (53.3) /14 (46.7)/0 (0)	2 (2.0)/44 (44.9)/51 (52.0)/1 (1.0)	0.163
Reconstruction, BI (%)/BII/RY/EG/DT	8 (40.0)/5 (25.0)/6 (30.0)/1 (5.0)/0 (0)	11 (36.7)/6 (20.0)/11 (36.7)/1 (3.3)/1 (3.3)	31 (31.6)/9 (9.2)/49 (50.0)/2 (2.0)/7 (7.1)	0.329
Combined resection, yes (%) Gall bladder/Spleen/Pancreas /Colon/Intestine/Renal (Partial)	0 (0) 0/0/0/0/0/0	1 (3.3) 1/0/0/0/0/0	19 (19.4) 9/7/3/1/0/1	0.013
Blood loss, ml, median (range)	35 (20–348)	25 (10–475)	25 (5–2540)	0.830 (S vs. Si)
				0.707 (Si vs. Xi)
				0.556 (Xi vs. S)
Intraoperative transfusion, yes (%)	0 (0)	1 (3.3)	1 (1.0)	0.538
No. of retrieved lymph nodes, median (range)	36 (21–84)	35 (10–81)	30 (10–103)	0.856 (S vs. Si)
				0.247 (Si vs. Xi)
				0.154 (Xi vs. S)
R classification R0^a^ (%)	20 (100)	29 (96.7)	96 (98.0)	0.715
**Postoperative complications**				
Overall complication^b^, ≥ grade II (%)	2 (10.0)	4 (13.3)	8 (8.2)	0.696
Overall complication, ≥ grade IIIa (%)	1 (5.0)	4 (13.3)	6 (6.1)	0.380
Reoperation, grade IIIb (%)	0 (0)	1^c^ (3.3)	1^d^ (1.0)	0.538
Mortality (%)	0 (0)	0 (0)	0 (0)	1.000
**Surgical complications**				
Anastomotic leakage, ≥ grade II (%)	0 (0)	2 (6.7)	2 (2.0)	0.285
Pancreatic fistula, ≥ grade II (%)	0 (0)	0 (0)	0 (0)	1.000
Intra-abdominal abscess, ≥ grade II (%)	0 (0)	0 (0)	3 (3.1)	0.458
Intra-abdominal bleeding, ≥ grade II (%)	2 (10)	0 (0)	0 (0)	0.002
Intra-luminal bleeding, ≥ grade II (%)	0 (0)	0 (0)	1 (1.0)	0.773
Ileus, ≥ grade II (%)	0 (0)	1 (3.3)	0 (0)	0.093
Cholecystitis, ≥ grade II (%)	0 (0)	0 (0)	0 (0)	1.000
Stenosis, ≥ grade IIIa (%)	0 (0)	0 (0)	0 (0)	1.000
Wound infection, ≥ grade II (%)	0 (0)	0 (0)	1 (1.0)	0.773
**Medical complications**				
Pneumonia, ≥ grade II (%)	0 (0)	0 (0)	1 (1.0)	0.773
Cardiovascular system, ≥ grade II (%)	0 (0)	0 (0)	0 (0)	1.000
Liver system, ≥ grade II (%)	0 (0)	0 (0)	0 (0)	1.000
Urinary system, ≥ grade II (%)	0 (0)	0 (0)	0 (0)	1.000
Thrombosis, ≥ grade II (%)	0 (0)	0 (0)	0 (0)	1.000

*DG* distal gastrectomy; *TG* total gastrectomy; *PG* proximal gastrectomy; *PAND* para-aortic nodal dissection; *BI* Billroth-I reconstruction; *BII* Billroth-II reconstruction; *RY* Roux-en-Y reconstruction; *EG* esophago-gastrostomy; *DT* double-tract reconstruction.

^a^Japanese Classification of Gastric Carcinoma.

^b^Surgical complications were classified into five categories according to the Clavien–Dindo classification.

^c^Laparoscopic surgery to repair the adhesive intestinal obstruction.

^d^Laparoscopic resection of anastomotic ulcer for delayed anastomotic ulcer.

### Postoperative complications

Table 3 summarizes postoperative complications. The incidence of complications higher than Clavien-Dindo grade II were defined as clinically significant. Postoperative complications of higher than grade II were observed in 2 patients (10%) in the S group (1 case of grade II and 1 case of grade IIIa), 4 patients (13.3%) in the Si group (3 cases of grade IIIa and 1 case of grade IIIb) and 8 patients (8.2%) in the Xi group (2 cases of grade II, 5 cases of grade IIIa and 1 case of grade IIIb). There was no significant difference. Reoperations were required for 1 patient (3.3%) in the Si group and for 1 patient (1.0%) in the Xi group (*P* = 0.538). One case of adhesive intestinal obstruction in the Si group required laparoscopic repair. One case in the Xi group had delayed anastomotic ulcer on gastroduodenostomy. The patient required laparoscopic resection of anastomotic ulcer with Roux-en-Y gastrojejunostomy. No patients had life-threatening complications requiring ICU-management (grade IV). The mortality rate in the three groups in our consecutive series was zero.

In comparison of each subtype of complication, no difference was observed in surgical complications including anastomotic leakage, pancreatic fistula, intra-abdominal abscess, intra-luminal bleeding, ileus, cholecystitis, stenosis, wound infection, and medical complications. Postoperative intra-abdominal bleeding was observed in two patients in the S group (1 case of grade II, 1 case of grade IIIa), the incidence rate was thus significantly higher than the other groups (*P* = 0.002).

## Discussion

We compared the surgical results of the da Vinci S/Si to the newer Xi during robotic gastric cancer surgery in 148 patients. The overall operation time in the Xi and Si groups was found to be 85 min shorter than in the S group. Operation time between the newer Xi and Si systems was not different. The duration of robot docking in Xi group was significantly shorter than in the S/Si groups. Intraoperative blood loss was similar in the three groups. The overall postoperative complication rate in the Xi group (8.2%) was lower than that of the S group (10%) and the Si group (13.3%), but the difference was not significant.

The operation time was significantly longer in the S group than in the other Si and Xi groups. Operation time in S group is biased by a different technological system but also by a different learning and skill curve. As a first generation system, surgeons had to face off docking issues and arms-fighting problems.

Advances in the newer da Vinci Xi system include an adapted user interface offering more assistance, with robotic setup and installation, a new vision architecture with chip-at-the-tip technology, torpedo-shaped robotic arms that are mounted on a rotation beam, longer instruments, and integrated energy device^8–10^. Although these technologies led to shortened docking time in the Xi group, neither overall operation nor console times were different to those in the Si group. Furthermore, the mechanical benefits of the Xi system did not lead to reduced intraoperative bleeding or postoperative complications. Postoperative intra-abdominal bleeding was observed in two cases only in the da Vinci S group, perhaps because the EndoWrist vessel sealing device (Vessel Sealer, Intuitive) could not be used during RG using the S system.

In rectal cancer surgery performed in the narrow pelvic cavity, the advanced technologies of the Xi system may be useful for safe total meso-rectal excision. Indeed, several studies have indicated that the use of da Vinci Xi is associated with a shorter operative time, reduced docking time, and higher full robotic resection rates than by da Vinci Si^8,11^. In robotic gastric cancer surgery performed in the wide space of the upper abdomen, however, the maneuverability advantages of the Xi system in comparison with the da Vinci Si are less clear. A previous study also demonstrated no difference in short-term surgical outcomes between the da Vinci Xi and da Vinci Si systems for RG^10^.

The median body mass index (BMI) of gastric cancer patients who participated in this study was 22. In this study conducted in Japan, which is dominated by patients with lower BMI than Western countries, the benefits of da Vinci Xi Surgical System could be underestimated, because thin patients have the clearer anatomy and lymph node dissection allows by definition comfortable dissection layers. In obese and overweight patients of Western countries, da Vinci Xi may be possible to offer a comfortable, time sparing gastric cancer surgery compared to Si System.

In this study, three cases (two in the Si group and one in the Xi group) were converted to laparoscopic or open surgery, the conversion rate was thus the same in the three groups. The cause of conversion to open surgery during RG using the da Vinci Xi was technical failure. The conversion rate of 0.7% to open surgery during RG was lower than previously reported^12,13^. The advantages of robotic surgery, including magnified 3D view and stable-movement forceps, allow precise dissectible layers and avoid injury to the adjacent organs.

This study has several limitations. It was a retrospective study without randomized controlled trial (RCT) and it was conducted in a single institution. Due to the small sample size, especially the number of surgical cases of the da Vinci S and Si systems, analyses would decrease statistical power. In addition, patients were allocated to the three groups according to sequential nature of the surgical system. Furthermore, we did not show the long-term oncological outcomes of patients who underwent RG, which might confirm the final impact of robotic gastric cancer surgery. A multi-center prospective RCT evaluating benefits including postoperative complications, quality of life, or more long-term outcomes in patients with GC treated with RG using the da Vinci Xi system is required.

This study verified the superiority of the newer robotic gastric cancer surgery and demonstrated good short-term surgical outcomes in S, Si and Xi groups, with low proportion of postoperative complications without mortality. However, the newer Xi system was not shown to provide significant intraoperative or early postoperative advantages over the Si system. Further studies are needed to examine the true benefits of the da Vinci Xi Surgical System regarding not only short-term surgical, but also long-term oncological outcomes.

## Materials and methods

### Patients

This single-center retrospective analysis of prospectively collected data was approved by the Institutional Review Board (IRB) at the Wakayama Medical University Hospital (WMUH). The committee that approved the research, confirmed that all research was performed in accordance with relevant guidelines/regulations. Written informed consent was obtained from all participants. The all research have been performed in accordance with the Declaration of Helsinki.

Between May 1, 2017, and January 31, 2021, 476 patients received radical gastrectomy for GC at WMUH. Of these, 150 underwent RG and the 303 patients received LG and the remaining 23 open gastrectomy. Patients with GC that underwent RG were included as part of a clinical trial (UMIN000027969/000031536). Among patients that underwent RG, we excluded one patient with GC in the remnant stomach after gastrectomy and one patient with cancer at the esophagogastric junction that required intrathoracic anastomosis. The remaining 148 consecutive patients were included in this retrospective study.

We began using RG in 2017 with the da Vinci S Surgical System. In January 2018, it was replaced by the Si and Xi systems and we subsequently performed RG procedures exclusively with the new systems. We used da Vinci Xi on Mondays and Si on Wednesdays. This study compares the short-term surgical outcomes of the RG using the da Vinci S, Si and Xi systems.

Tumor stage was classified by the International Union Against Cancer TNM criteria, Eighth Edition^14^. All surgical and medical complications and mortality events were documented. Postoperative complications were analyzed according to Clavien-Dindo classification^15^. Complications higher than grade II were considered to be clinically significant. Surgical complications were confined to events that occurred within 90 days after surgery; these included anastomotic leakage, pancreatic fistula, intra-abdominal abscess, intra-abdominal bleeding, intraluminal bleeding, ileus, cholecystitis, anastomotic stenosis, and wound infection. Medical complications included pulmonary, cardiovascular, liver, urinary and thrombosis events. Reoperation cases (= grade IIIb) were defined as any reoperation connected with any surgery-related complications. Mortality was defined as any death that occurred during the hospital stay.

Operation time was defined as the time from the skin incision to skin closure, docking time was the time from the trocar placement to being ready to start the console, and console time was the overall surgery time at the console.

### Surgical procedures

Details of the RG procedures performed at WMUH have been previously described^1,2^. All RG procedures were performed using da Vinci S, Si or Xi Surgical System with four articulating robotic arms; a central arm for a 30° rigid endoscope, a first arm for monopolar scissors, a second arm for fenestrated bipolar forceps, and a third arm for Cadiere forceps. One additional port for assisting forceps was placed at the right umbilical level. Robotic ultrasonically activated device (USAD) does not have wrist-like motion, and does not, therefore, have robotic articulated function. For these reasons, we did not use robotic USAD. We performed lymph node dissection using a monopolar scissors (da Vinci S, Si and Xi Surgical System) and a Vessel Sealer (da Vinci Si and Xi). D1 or D1 + dissection was applied for clinical stage IA tumors, while D2 or D2 + para-aortic nodal dissection was performed for tumors higher than clinical stage IB^16^. Dissection of lymph node station 14v was optional, but an omentectomy was essential for tumors higher than clinical T2^16^. The greater omentum was resected up to the inferior portion of the spleen. The left gastroepiploic vessels were dissected at the point before the first branch (nos. 4d, 4sb). After completion of omentectomy, the root of the right gastroepiploic vein and artery were isolated and transected (no. 6). The root of the right gastric artery was isolated in the hepatoduodenal ligament and transected (no. 5). The lesser omentum along the liver edge to the esophagogastric junction was resected. The peri-gastric lymph nodes were dissected along the upper lesser curvature up to the esophagogastric junction (nos. 1 and 3). For robotic D1 + lymphadenectomy, the lymph nodes around the celiac trunk (no. 9) were dissected, and the root of the left gastric vein and artery were isolated and transected (no. 7), and successively, the lymph nodes along the common hepatic artery were dissected (no. 8a). For robotic D2 lymph node dissection, the lymph nodes along the proper hepatic artery (no. 12a) and along the splenic artery (no. 11) were also dissected. Lymph node dissection was completed intra-corporeally. In RG using articulating forceps, lymphadenectomy without touching the pancreas was possible. Intracorporeal anastomosis using linear staplers, such as gastroduodenostomy, gastrojejunostomy, or esophagojejunostomy was performed^17–19^. When an incision exceeding 10 cm was required for the control of intraoperative complications or tumor extension, the procedure was defined as a conversion to open surgery.

### Statistical examinations

SPSS version 24.0 (SPSS, Chicago, IL) was used for all statistical analyses. Quantitative results are expressed as medians and ranges. Statistical comparisons between three groups were performed using chi-squared statistics, in the case of two groups it was by Mann–Whitney U test. A *P* < 0.05 was considered to be significant.

### Human rights statement and informed consent

This study was approved by the Institutional Review Board and the Ethics Committee of Wakayama Medical University. The study protocol was registered at the University Hospital Medical Information Network (UMIN000027969/000031536).
